# Attitude of Interns towards Family Medicine as a Career in a Tertiary Care Hospital

**DOI:** 10.31729/jnma.4634

**Published:** 2019-10-31

**Authors:** Anu Kushwaha, Anuj Raj Kadel

**Affiliations:** 1 Department of Emergency Medicine and General Practice, Kathmandu Medical College Teaching Hospital Sinamangal, Kathmandu, Nepal; 2 Kathmandu Medical College Teaching Hospital, Duwakot, Bhaktapur, Nepal

**Keywords:** *attitude*, *career*, *family medicine*

## Abstract

**Introduction:**

Family medicine is a relatively overlooked specialty in Nepalese medical education. It is unclear how many interns are actually interested in Family medicine as a career and how many non-medical individuals are aware of its existence. The aim of this study is to assess information, expectation and perception among interns regarding family medicine and its choice as a career.

**Methods:**

This is a descriptive-cross sectional study carried out in Kathmandu Medical College Teaching Hospital from July 2018 to December 2018. Whole sampling was done. All interns of Kathmandu Medical College Teaching Hospital posted in different departments during the time period was the inclusion criteria. Chronic absentees was the exclusion criteria. Factor like age, sex, their residency of choice, and whether they consider family medicine as a potential career were considered. Data was entered in with Statistical Package for Social Services version 16 and necessary calculations were done.

**Results:**

Thirty three interns preferred internal medicine as their career of choice, 20 preferred surgery while only three preferred in family medicine. Eighty eight interns, including the three who had family medicine as their career of choice, said that they could consider family medicine as a potential career. Eighty eight out of the 100 interns in the study mentioned that their family had knowledge that a specialty called family medicine existed.

**Conclusions:**

Despite being the career of choice of only a few, majority considered family medicine as a potential career for them. And the knowledge about existence of family medicine was high among families of interns.

## INTRODUCTION

The field of family medicine is emerging as a cornerstone for providing comprehensive, quality care to a diverse population.^1^ Even then, there are very few number of interested young doctors to pursue family medicine. One scenario in context of Nepal might be for a family medicine specialist to be placed in a primary care center. More often than not, the development of primary health care system is hindered by insufficiency of family medicine physicians. Moreover, most medical colleges in Nepal lack the course in the family medicine.

Choosing a medical career is a difficult process which depends on multiple factors like personality, educational environment and perception of professional practice. Some specialties are often overlooked due to the lack of insight into their importance and responsibilities. Adequate studies have not been done in this regard. The aim of this study is to enquire how many intern doctors considered family medicine as a future career.

## METHODS

This is a descriptive cross sectional study conducted in Kathmandu Medical College Teaching Hospital in year 2018 from July to December. Whole sampling was done. The study was started after ethical clearance from Institutional Research Committee of Kathmandu medical college teaching hospital. All the interns were included in the study however, excluded interns were those who could not participate due to absence. Besides the demographic profile of interns, they were asked about their career of choice and whether they could potentially consider family medicine as a career. The data collected through questionnaire were then entered in with Statistical Package for Social Services (SPSS) version 16 and necessary calculations were done.

## RESULTS

Out of the 100 interns enrolled into this study, 62 were below 25 years of age and 38 were 25 years of age and above. There were 65 males and 35 females. 33 interns showed interest in internal medicine, 20 in surgery and 17 orthopedic and 17 in others while only three had interest in family medicine.

**Table 1:** Frequencies of different variable.

Variables		n (%)
Age	25>	62 (62)
	25=<	38 (38)
Sex	Male	65 (65)
	Female	35 (35)
Address	Inside Valley	73 (73)
	Outside Valley	27 (27)
Preferred Residency	Medicine	33 (33)
	Surgery	20 (20)
	Orthopedic Surgery	17 (17)
	Obstetrics & Gynecology	10 (10)
	Family Medicine	3 (3)
	Others	17 (17)

Among the 33 interns who preferred internal medicine, 30 (91%) considered family medicine as a potential career. Similarly, 17 (85%) out of 20 who preferred surgery, 17 (100%) out of 17 who preferred orthopedics, 3 (100%) out of 3 who preferred family medicine, 8 (80%) out of 10 who preferred Obstetrics and Gynecology and 13 (76%) out of 17 who preferred other career than those listed could see themselves working as a family medicine physician.

**Figure 1:**
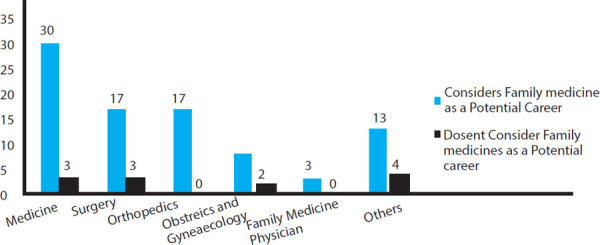
Preferred Career and Family Medicine as a potential career.

## DISCUSSION

Studies relating to interns doctors and their career is rarely done in Nepal. This is the reason why we decided to study the subject. In our study only 3% of interns were interested in Family medicine. Such a low number of interested students were present in a study in Ghana,^2^ the reason of which was stated to be an inadequate understanding of the speciality.^2^ However, in most other countries the percentage of interested was higher^3-6^ being the highest in developed countries like Canada (52%).^3^ The respect for family medicine physicians was present across the board. However, even though some students find family medicine appealing, it is regarded as a career of low interest and prestige.^7^

In a study conducted in Saudi Arabia, it was observed that medical students generally pursue careers in internal medicine, surgery, pediatrics, emergency medicine and family medicine. Their study findings suggested that the lifestyle and personal priorities of the medical residents are prime factors responsible for their choice of specialty.^1^ In addition, a study conducted in London concluded that the most important influence on students’ attitude was their direct personal experience of general practice and that 5th year students were more likely to become interested in General physcian than 1st year.^8^ Clinical rotation experience was found in some studies to have a positive influence on the future specialty choice.^9^ In some countries shorter working hours for a family medicine physician was an attractive feature, but the hours were longer in developed countries like US and Canada probably due to level of awareness about family medicine among the general population of those countries and that patient are taken to ER unnecessarily in developing countries.^9^ The influence of family and friends plus a direct personal experience played an important role in our study; similar to a Japanese study.^10^ Training in family medicine can create a positive attitude towards primary health care.^11^ In Nepal, where the rural areas are in scarcity of doctors, family medicine physicians would be of immense value. But the number of interested students to pursue family medicine is not encouraging. Several steps could be taken to mitigate this. Firstly, several studies have shown that students generally make their mind up about their career choice around the time they have clinical rotations so introducing primary health care rotation could have a positive influence.^6,8,9^ As was shown by our study, a good number of family of interns were aware about family medicine, which could be taken as In another study done in Nepal, serving the sick, personal interest, and social prestige were most significant influencing factors in choosing a specialty. Course availability was also a factor. To attract doctors to work in rural areas most respondents affirmed the need for a good salary, infrastructure and facilities, scholarships and career development opportunities.^12^ Interactions with interest groups in family medicine (IGFM) has shown to have a positive influence towards choosing family medicine as a specialty.^13^ There is an important role of orientations as evidenced by a study done in Egypt where the percentage of House officers who will choose family medicine as a career increased from 15.8% pre-orientation to 50.4% post-orientation.^4^

## CONCLUSIONS

Although majority of interns had other specialties as their first choice, they showed a positive attitude towards family medicine as a potential career. A large proportion of family related to the interns were familiar to family medicine as a specialty. Thus, there is a good scope for development of family medicine in Nepal.

## Conflict of Interest:

None.

## References

[1] Alyousefi NA (2017). Knowledge and attitude of Saudi medical students towards the family medicine speciality during their family medicine course and its effect on their career plans: A comparative study. Biomed Res-India.

[2] Essuman A, Anthony-Krueger C, Ndanu TA (2013). Perceptions of medical students about family medicine in Ghana. Ghana Med J..

[3] Scott I, Gowans M, Wright B, Brenneis F, Banner S, Boone j (2011). Determinants of choosing a career in family medicine. CMA..

[4] Elkhawaga G, Nernard M, El-Gilany (2015). House officers attitude towards family medicine and its choice as a career in Egypt. A H. Fam Prac..

[5] Ashraf I, Chan WWT, Prasad RK, Kubendra M, Hemavathy D, Prasad S (2018). Family medicine: perception and attitude among Indian medical students. J of Family Med Prim Care..

[6] Ryuichi K, Daisuke N, Yoshihisa K, Tomo K, Nobuyuki O, Kumagi Teru, Masanori A (2016). Factors associated with the choice of general medicine as a career among Japanese medical students, Medical Education Online..

[7] Olid A S, Zurro A M, Villa J J, Hijar A M, Tuduri X M, Puime AO (2012). Medical students’ perceptions and attitude about family medicine: a qualitative research synthesis. BMC Med Educ..

[8] Henderson E, Berlin A, Fuller J (2002). Attitude of medical students towards the general practice and gereral practitioners. Br J Gen Pract..

[9] Alshahrani M, Dhafery B, Al Mulhim M, Alkhadra F, Al Bagshi D, Bukhamsin N (2014). Factors influencing Saudi medical students and interns’ choice of future specialty: a self-administered questionnaire. Adv Med Educ Pract..

[10] Kenle l, Tahara M, Murata A, Komiyama M, Onishi H (2014). Factor associated to the career choice of family medicine among Japanese physician: the down of new era. Asia Pacific family medicine.

[11] Al-Dawood KM, Elzubier AG (2002). ATTITUDES OF MEDICICAL TOWARDS THE PRACTICE OF PRIMARY HEALTH CARE. J Family Community Med..

[12] Hayes B W, Shakya R (2013). Career choices and what influences Nepali medical students and young doctors: a cross-sectional study. Hum Resour Health.

[13] Kerr JR, Seaton MB, Zimcik H, McCabe J, Feldman K (2008). The impact of interest: how do family medicine interest groups influence medical students?. Can Fam Physician..

